# Synaptic connectivity of the TRPV1-positive trigeminal afferents in the rat lateral parabrachial nucleus

**DOI:** 10.3389/fncel.2023.1162874

**Published:** 2023-03-30

**Authors:** Su Bin An, Yi Sul Cho, Sook Kyung Park, Yun Sook Kim, Yong Chul Bae

**Affiliations:** Department of Anatomy and Neurobiology, School of Dentistry, Kyungpook National University, Daegu, Republic of Korea

**Keywords:** synaptic connectivity, trigeminal, nociceptive, lateral parabrachial nucleus, ultrastructure

## Abstract

Recent studies have shown a direct projection of nociceptive trigeminal afferents into the lateral parabrachial nucleus (LPBN). Information about the synaptic connectivity of these afferents may help understand how orofacial nociception is processed in the LPBN, which is known to be involved primarily in the affective aspect of pain. To address this issue, we investigated the synapses of the transient receptor potential vanilloid 1-positive (TRPV1+) trigeminal afferent terminals in the LPBN by immunostaining and serial section electron microscopy. TRPV1 + afferents arising from the ascending trigeminal tract issued axons and terminals (boutons) in the LPBN. TRPV1+ boutons formed synapses of asymmetric type with dendritic shafts and spines. Almost all (98.3%) TRPV1+ boutons formed synapses with one (82.6%) or two postsynaptic dendrites, suggesting that, at a single bouton level, the orofacial nociceptive information is predominantly transmitted to a single postsynaptic neuron with a small degree of synaptic divergence. A small fraction (14.9%) of the TRPV1+ boutons formed synapses with dendritic spines. None of the TRPV1+ boutons were involved in axoaxonic synapses. Conversely, in the trigeminal caudal nucleus (Vc), TRPV1+ boutons often formed synapses with multiple postsynaptic dendrites and were involved in axoaxonic synapses. Number of dendritic spine and total number of postsynaptic dendrites per TRPV1+ bouton were significantly fewer in the LPBN than Vc. Thus, the synaptic connectivity of the TRPV1+ boutons in the LPBN differed significantly from that in the Vc, suggesting that the TRPV1-mediated orofacial nociception is relayed to the LPBN in a distinctively different manner than in the Vc.

## Introduction

The synaptic connectivity of the primary sensory afferent terminals differs according to the type of the parent primary afferent and its target (Park et al., 2016, 2019). For example, the terminals of the Aδ and the peptidergic C fibers differ in the degree of synaptic divergence and presynaptic modulation in the spinal dorsal horn (SDH; Alvarez et al., 1992, 1993). In addition, the terminals of the Aβ fibers have different synaptic connectivity in the functionally different trigeminal principal and oral nuclei (Bae et al., 1994, 2000). This suggests that the sensory information conveyed *via* primary afferents is transmitted and processed differently depending on the type of the primary afferent and the particular target nucleus.

The lateral parabrachial nucleus (LPBN), located in the dorsolateral pons, receives nociceptive input from the orofacial- and other body areas, and relates it to several brain regions, such as the central amygdala, the hypothalamus, and the bed nucleus of stria terminalis, which are known to be involved in the control of instinctual behavior and emotions (Rodriguez et al., 2017; Schier and Spector, 2019). Multiple previous studies have revealed that LPBN receives dense, indirect (polysynaptic) nociceptive input relayed *via* second-order neurons in the trigeminal caudal nucleus (Vc: medullary dorsal horn) and SDH (Feil and Herbert, 1995; Saito et al., 2017). However, some more recent studies using neural tracing and trigeminal rhizotomy also showed that LPBN receives direct (monosynaptic) nociceptive input from the orofacial area by way of the trigeminal primary afferents (Cavanaugh et al., 2011; Panneton and Gan, 2014; Rodriguez et al., 2017; Uddin et al., 2021).

We previously reported a distinct synaptic connectivity of the transient receptor potential vanilloid 1-positive (TRPV1+) axon terminals in the Vc, which is known to be involved in the perceptual, discriminative, and autonomic aspects of pain (Yeo et al., 2010). However, little is known about the synaptic connectivity of the TRPV1+ axon terminals in the LPBN; this may help understand how orofacial nociception is processed in the LPBN, which is known to be involved primarily in the affective aspect of pain (Han et al., 2015; Rodriguez et al., 2017).

To address this, we analyzed the synaptic connectivity of the TRPV1+ trigeminal afferent terminals in the LPBN using light- and electron microscopic (EM) immunohistochemistry and serial section electron microscopy.

## Materials and methods

### Animal and tissue preparation

The laboratory animal care and all experimental procedures were performed in accordance with the National Institute of Health guidelines and were approved by the Kyungpook National University Intramural Animal Care and Use Committee.

A total of eight 9-week-old male Sprague–Dawley rats weighing 300–320 g were used for this study: three and five rats were used for light microscopic (LM) and EM immunohistochemistry, respectively. The rats were deeply anesthetized with a mixture of ketamine (80 mg/kg) and xylazine (10 mg/kg) administered intraperitoneally and were perfused transcardially with 80 ml of heparinized saline, followed by 300 ml of a freshly prepared fixative: Fixative was 4% paraformaldehyde (PFA) in 0.1 M phosphate buffer (PB, pH 7.4) for LM immunohistochemistry and was 0.01% glutaraldehyde and 4% PFA in 0.1 M PB (pH 7.4) for EM immunohistochemistry. The brainstem and the trigeminal ganglia (TG) were removed and postfixed in the same fixative for 2 h at 4°C. Then, for LM, tissues were immersed in 30% sucrose in PB at 4°C overnight and 40-μm thick sections were cut on a cryotome and collected in PB at 4°C. For EM, 60-μm thick sections were cut on a Vibratome and immersed in 30% sucrose in PB at 4°C overnight.

### Light microscopic immunohistochemistry

For LM, sections were stained for TRPV1 with immunoperoxidase. Briefly, sections of brain stem and TG were treated with 50% ethanol for 30 min, to improve antibody penetration into the tissue, with 3% H_2_O_2_ in PB for 10 min, to block the endogenous peroxidases, and with 10% normal donkey serum (NDS; Jackson ImmunoResearch, West Groove, PA, USA) in PB for 30 min to mask secondary antibody binding sites. Then, the sections were incubated with 10% NDS and with a goat anti-TRPV1 antibody (AF3066, R&D systems, Minneapolis, MN, USA) at a 1:200 dilution in phosphate-buffered saline (PBS; 0.01 M, pH 7.4) for 2 h at room temperature. Biotin-Avidin-Peroxidase labeling was performed by incubation with ExtrAvidin peroxidase (1:5,000 in PBS; Sigma-Aldrich, St. Louis, MO, USA) for 1 h. Immunoperoxidase was revealed using the nickel-intensified 3, 3′-diaminobenzidine tetrahydrochloride (Ni-DAB) protocol. The sections were mounted on slides, examined on a Zeiss Axioplan 2 microscope (Carl Zeiss, Gottingen, Germany) and digital images were obtained with an Exi camera (Q-Imaging Inc., Surrey, CA, USA).

### Electron microscopic immunohistochemistry

Sections of the LPBN, the Vc, and the TG were frozen on dry ice for 20 min and rapidly thawed in 0.01 M phosphate-buffered saline (PBS, pH 7.4) to enhance antibody penetration into the tissue, and incubated with 3% H_2_O_2_ for 10 min to suppress endogenous peroxidases. Then, the sections were incubated with 10% NDS for 30 min and with goat the anti-TRPV1 antibody at a 1:100 dilution in PBS overnight. On the next day, the sections were incubated with 2% NDS for 10 min and then with biotinylated donkey anti-goat antibody (Jackson ImmunoResearch) at a 1:200 dilution in PBS for 2 h. After washing in PBS, the sections were incubated with ExtrAvidin peroxidase (1:5,000) for 1 h. The immunoperoxidase was visualized by Ni-DAB.

After washing in PB, sections were treated with 1% osmium tetroxide in PB for 1 h, dehydrated in a serial dilution of ethanol, flat-embedded in Durcupan ACM resin (Fluka, Buchs, Switzerland) between strips of Aclar film (EMS, Hatfield, PA, USA), and then cured at 60°C. After 48 h, the film was stripped and the embedded sections were observed under light microscope. Chips of approximal size of 1 × 1 mm containing many TRPV1+ boutons in the LPBN and in the superficial lamina of the Vc were cut out and glued onto blank resin blocks with cyanoacrylate. Thin sections were cut and mounted serially on the Formvar coated single-slot nickel grids. The grids were stained with uranyl acetate and lead citrate, and examined with a Hitachi H-7500 electron microscope (Hitachi, Tokyo, Japan) at 80 kV.

For analysis of the synaptic connectivity of the TRPV1+ boutons in the LPBN, electron micrographs were taken from every serial thin section through individual TRPV1+ axon terminals at a final magnification X30,000. Non-serial thin sections of the TRPV1+ axon terminals in the Vc were also studied for differences in the synaptic connectivity of the TRPV1+ axon terminals in LPBN and in Vc. Images was captured with a Digital Micrograph software driving a cooled CCD camera (SC1000; Orius; Gatan, Pleasanton, CA, USA) attached to the microscope, and saved as TIFF files. The brightness and contrast of the images were adjusted in Adobe Photoshop CS5.1 (Adobe Systems Inc., San Jose, CA, USA). Inter-animal variability in frequency of occurrence of different types of contacts per TRPV1+ bouton was insignificant (one-way ANOVA), and the data could be pooled for analysis. Values (mean ± SD) in the frequency of occurrence (%) of TRPV1+ boutons according to the number of postsynaptic dendrites were calculated from 5 animals (*n* = 5) in the LPBN and 3 animals in the Vc (*n* = 3, Yeo et al., 2010). Values (mean ± SD) in the frequency of occurrence (numbers) of different types of synaptic contacts per TRPV1+ bouton are were calculated from 43 (*n* = 43, in 5 animals) and 76 (*n* = 76, in 3 animals, Yeo et al., 2010) boutons in the LPBN and Vc, respectively.

### Immunohistochemical controls

**FIGURE 1:**
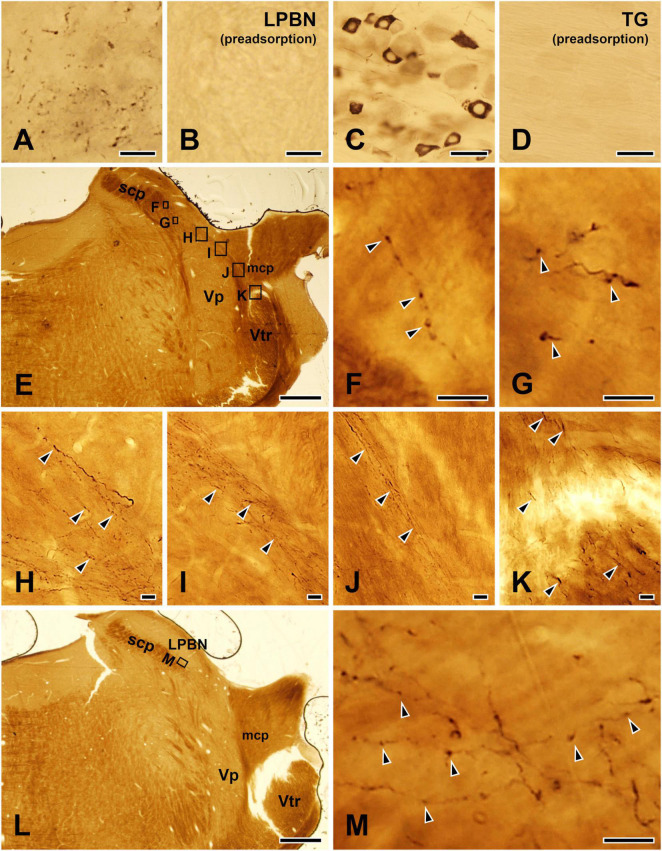
Light micrographs showing immunostaining for TRPV1 in the lateral parabrachial nucleus (LPBN: **A,B**) and the trigeminal ganglion (TG: **C,D**), the TRPV1+ axons and terminals in the LPBN arising from the ascending trigeminal tract **(E–K)**, and examples of TRPV1+ axon terminals in the LPBN that were further analyzed by electron microscopy **(L,M)**. **(A–D)** Immunohistochemical staining for TRPV1 in the LPBN **(A,B)** and TG **(C,D)**. The TRPV1 immunostaining in the axons in the LPBN and neurons in the TG was completely abolished by pre-adsorption with a blocking peptide (10 μg/ml), confirming the specificity of the TRPV1 antibody. **(E–K)** Light micrographs showing that the TRPV1+ axons and terminals in the LPBN **(E–G)** arise from the ascending trigeminal tract (Vtr: **K**). These axons **(K)** course along the medial edge of the middle cerebellar peduncle (mcp: **J**) and the dorsal border of the trigeminal principal nucleus (Vp: **H,I**) and issue axon collaterals and terminals in the LPBN **(F,G)**. **(L,M)** Examples of TRPV1+ axons and terminals in the LPBN that were further studied by electron microscopy. **(F–K,M)** Are enlargements of the boxed areas in panels **(E,L)**, respectively. Arrowheads indicate TRPV1+ axons and terminals. scp, superior cerebellar peduncle. Scale bars = 10 μm in panels **(A,B,F–K,M)**, 50 μm in panels **(C,D)** and 500 μm in panels **(E,L)**.

To control for specificity of the TRPV1 antibody, sections of LPBN and TG were incubated with the TRPV1 antibody, which was pre-adsorbed with a TRPV1 blocking peptide (PEP094, ThermoFisher Scientific, Waltham, MA, USA) at a final concentration of 10 μg/ml. Specific immunostaining for TRPV1 was completely abolished by pre-adsorption with the TRPV1 blocking peptide (Figures 1A–D).

## Results

At light microscopy, multiple TRPV1-immunopositive (+) axons apparently arising from the ascending trigeminal tract, coursed along the medial edge of the middle cerebellar peduncle, the dorsal border of the trigeminal principal nucleus, and issued many fibers and en passant and terminal boutons, in the LPBN, indicating that the TRPV1+ axon terminals in the LPBN arise from ascending trigeminal tract that is mostly composed of trigeminal primary sensory afferents (Figures 1E–M).

**FIGURE 2:**
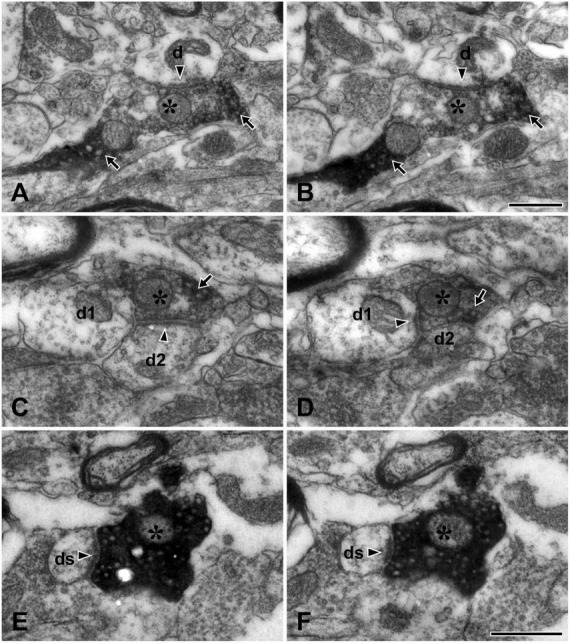
Electron micrographs of adjacent thin sections (**A** and **B**, **C** and **D**, **E** and **F**, each pair about 200 nm apart) in the lateral parabrachial nucleus (LPBN) showing TRPV1+ boutons (asterisks) forming synapse with one dendrite (d in panels **A,B**), two dendrites (d1 and d2 in panels **C,D**) and with a dendritic spine (ds in panels **E,F**). The TRPV1+ bouton (asterisk) can be identified by the presence of electron-dense immunoreaction product (arrow) within the axoplasm. Arrowheads indicate synapses. Scale bars = 500 nm in panel **(B)** (also applies to panel **A**) and **(F)** (also applies to panels **C–E**).

At electron microscopy, the TRPV1+ axons and terminals (boutons) could be easily identified by the presence of electron-dense immunoreaction product within their axoplasm. The section profiles of the TRPV1+ boutons were usually round or slightly elongated in shape and those with glomerular or scalloped shape were rare. They contained round vesicles and typically formed synaptic contacts of asymmetric type with small- or medium-sized dendritic shafts and/or spines which could be identified by the presence of fuzzy cytoplasm and no mitochondria or no microtubule; those forming synaptic contacts with somata or proximal dendrites were rare (Figure 2).

In the present study, we analyzed the synapses of a total of 43 TRPV1+ boutons in the LPBN reconstructed from serial thin sections: The large majority (82.6 ± 9.3%, mean ± SD) of the TRPV1+ boutons formed a synaptic contact with a single dendrite (Figures 2A, B), and a few (15.7 ± 5.7%) with two dendrites (Figures 2C, D). Only one of the 43 terminals studied (1.7 ± 3.7%) formed synaptic contacts with three dendrites. Most (86.8 ± 10.6%) TRPV1+ boutons formed synaptic contact with dendritic shafts. Only a small fraction (14.9 ± 10.3%) of the TRPV1+ boutons formed synaptic contacts with dendritic spines (Figures 2E, F). TRPV1+ boutons receiving contacts from other terminals (typically axoaxonic synapses of symmetric type from boutons containing pleomorphic vesicles) were not observed (Figure 2 and Table 1).

**TABLE 1:** Frequency of occurrence (%) of TRPV1+ boutons according to the number of postsynaptic dendrites, and numbers of different types of synaptic contacts per TRPV1+ bouton in the lateral parabrachial nucleus (LPBN) and trigeminal caudal nucleus (Vc).

Region	No. of boutons examined	No. of postsynaptic dendrites							Type of postsynaptic profiles			Presynaptic endings (p-endings)
		**1**	**2**	**3**	**4**	**5**	**6**	**7**	**Dendritic shafts**	**Dendritic spines**	**Total number of dendrites**	
**LPBN**	43	82.6 ± 9.3* (35/43)	**15.7 ± 5.7** (7/43)	1.7 ± 3.7* (1/43)	0.0 ± 0.0* (0/43)	0.0 ± 0.0* (0/43)	0.0 ± 0.0* (0/43)	**0.0 ± 0.0** (0/43)	**1.0 ± 0.6** *	**0.2 ± 0.4** *	**1.2 ± 0.5** *	**0.0 ± 0.0** *
**Vc**	76	51.3 ± 3.0* (39/76)	**22.4 ± 2.8** (17/76)	7.9 ± 0.2* (6/76)	9.2 ± 2.4* (7/76)	3.9 ± 0.1* (3/76)	3.9 ± 0.1* (3/76)	**1.3 ± 2.2** (1/76)	**1.5 ± 1.3** *	**0.6 ± 0.8** *	**2.1 ± 1.5** *	**0.1 ± 0.4** *

Values are mean ± SD. “n” in parentheses indicates the number of TRPV1+ boutons/total number of TRPV1+ boutons examined. *Indicates significant difference between LPBN and Vc (Unpaired Student t-test, *p* < 0.05). Data on Vc are from pervious our study (Yeo et al., 2010).

**FIGURE 3:**
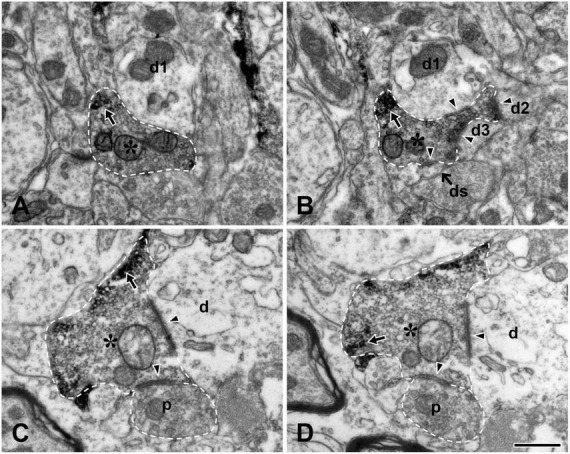
Electron micrographs of adjacent thin sections (**A** and **B**, **C** and **D**, each pair about 200 nm apart) in the trigeminal caudal nucleus (Vc). **(A,B)** A TRPV1+ bouton (asterisk) makes a synaptic contact with 3 dendritic shafts (d1–d3) and one dendritic spine (ds). **(C,D)** A TRPV1+ bouton (asterisk) makes a synaptic contact with one dendritic shaft (d) and receives an axoaxonic synapse from a presynaptic ending containing pleomorphic vesicles (p). Arrows indicate electron dense TRPV1 immunoreaction product. Arrowheads indicate synapses. TRPV1+ boutons and presynaptic ending are outlined with a dashed line. Scale bar = 500 nm in panel **(D)** (also applies to panels **A–C**).

We also examined the ultrastructure of the TRPV1+ boutons in the Vc in non-serial thin sections and confirmed our previous findings (Yeo et al., 2010) in the Vc, thus, TRPV1+ boutons frequently formed complex synaptic arrangements with three or more dendrites and those receiving axoaxonic synapses were frequently observed (Figure 3). In addition, we compared synaptic connectivity of the TRPV1+ boutons in the LPBN with that in the Vc which was reported in our previous study (Yeo et al., 2010): Frequency of TRPV1+ boutons that form synapse with one postsynaptic dendrite was significantly higher in the LPBN than Vc (82.6 ± 9.3 vs. 51.3 ± 3.0, *p* < 0.05, unpaired student *t*-test). Whereas frequency of TRPV1+ boutons forming synapse with 3 dendrites is significantly lower in the LPBN than Vc (1.7 ± 3.7 vs. 7.9 ± 0.2, *p* < 0.05, unpaired student *t*-test). Furthermore, a considerable fraction (18.4%) of TRPV1+ boutons in the Vc, but none in the LPBN, formed complex synaptic arrangement with 4 or more postsynaptic dendrites. Number of dendritic spine per TRPV1+ bouton was significantly fewer in the LPBN than Vc (0.2 ± 0.4 vs. 0.6 ± 0.8, *p* < 0.05, unpaired student *t*-test). Total number of postsynaptic dendrites per TRPV1+ bouton was also significantly fewer in the LPBN than Vc (1.2 ± 0.5 vs. 2.1 ± 1.5, *p* < 0.05, unpaired student *t*-test, Table 1).

## Discussion

The main finding of the present study is that virtually all TRPV1+ boutons in the LPBN establish simple synaptic contacts with 1–2 postsynaptic dendrites, and do not participate in complex synaptic arrangements with 4 or more dendrites and other synaptic terminals, which are frequently observed in the Vc, suggesting that TRPV1-mediated orofacial nociception is relayed in the LPBN in a distinctly different manner than in the Vc.

### All TRPV1+ boutons in the LPBN show simple synaptic connectivity

That TRPV1+ fibers apparently arising from the ascending trigeminal tract terminated in the LPBN (indicating that the TRPV1+ terminals in the LPBN are of primary sensory origin) is consistent with the studies using TRPV1-Cre mice (Cavanaugh et al., 2011; Rodriguez et al., 2017), trigeminal rhizotomy (Panneton and Gan, 2014), and neural tracing (Uddin et al., 2021) that report a direct projection of trigeminal primary nociceptive afferents to the LPBN. Studies using intra-cellular and intra-axonal injections of neural tracer showed that a single presynaptic axon terminal does not simultaneously contact two or more dendrites of the same neuron, suggesting that when a single axon terminal contacts two or more dendrites, each postsynaptic dendrite belongs to a separate neuron (Yabuta et al., 1996; Yoshida et al., 2001).

All TRPV1+ boutons in the LPBN formed simple synaptic contacts with one, rarely two, dendrites. This suggests that, at a single bouton level, TRPV1-mediated orofacial nociception is transmitted to one or two postsynaptic neurons that may project to specific brain regions with a small degree of synaptic divergence. This pattern of connectivity is very different from that in the Vc. Thus, a considerable fraction (∼26%) of TRPV1 + boutons form complex synaptic contacts with 3–7 dendrites in the Vc, and total number of postsynaptic dendrites per TRPV1 + bouton is also 1.8 times higher in the Vc (Yeo et al., 2010) than LPBN, suggesting that the TRPV1-mediated orofacial nociceptive signal may spread to multiple postsynaptic neurons in the Vc thus giving rise to a divergent afferent system to multiple brain regions. The different synaptic connectivity of the TRPV1+ boutons in the LPBN and the Vc can be related with the functional differences of these two regions: Neurons in the LPBN project to the central amygdala, the hypothalamus, and the bed nucleus of the stria terminalis, which are involved primarily in the affective aspect of pain (Rodriguez et al., 2017; Schier and Spector, 2019), whereas Vc, *via* various types of neurons including neurons projecting to thalamus and LPBN and interneurons, is connected to various brain regions, which are involved in pain perception as well as the emotional, the autonomic, and the reflexive motor responses to pain (Shigenaga et al., 1983; Iwata et al., 1992; Spike et al., 2003; Al-Khater et al., 2008).

Under certain pathological conditions, dendritic spines exhibit dynamic plastic changes in their size, and the number and size of their postsynaptic densities containing neurotransmitter receptors, leading to alterations of synaptic strength (Baczynska et al., 2021; Meldolesi, 2022). For example, following peripheral inflammation and nerve injury, the density and size of the dendritic spines of SDH neurons increase, which contributes to their hyperexcitability (Matsumura et al., 2015; Benson et al., 2020). Similarly, under pathological conditions, the nociceptive neurons in the Vc show extensive neuroplastic changes, such as increase in neuronal activity and decrease in activation threshold, which may contribute to central sensitization and hyperalgesia (Chiang et al., 1999, 2005; Wang et al., 2018). In the present study, about 14% of the TRPV1+ boutons in the LPBN formed synapses with dendritic spines, a much smaller fraction than that of TRPV1+ boutons (∼43%) and of nociceptive tooth pulp afferent boutons (∼60%) that form synapses with dendritic spines in the Vc (Bae et al., 2003; Yeo et al., 2010). In addition, number of dendritic spines per TRPV1+ bouton was much fewer in the LPBN than Vc. This difference between LPBN and Vc is analogous to the difference between the frequency of synapses of the tooth pulp afferents and the rostral nucleus of solitary tract afferents with dendritic spines in their respective functionally different target nuclei (Bae et al., 2003; Park et al., 2022). It is consistent with the idea that under pathological conditions, the change in synaptic strength and postsynaptic neuron excitability through spine plasticity can be less pronounced in the LPBN than in the Vc, thus affecting the emotional aspect of pain less than the pain perception *per se*. Further functional study in the LPBN and Vc during pathologic condition is needed to support the idea.

### TRPV1+ boutons in the LPBN do not participate in axoaxonic synapses

Multiple EM studies have shown that the frequency of axoaxonic synapses involving the same type of axon differs among functionally different target nuclei. For example, we reported that the terminals of large myelinated Aβ afferents (Bae et al., 1994) and the tooth pulp afferents (Bae et al., 2003) participate more frequently in axoaxonic synapses in the trigeminal principal nucleus than in the trigeminal oral and caudal nuclei. In the present study, none of the TRPV1+ boutons in the LPBN participated in axoaxonic synapses, in contrast to the considerable fraction (13%) of TRPV1+ boutons that participated in axoaxonic synapses in the Vc (Yeo et al., 2010). This suggests that, in the LPBN, TRPV1-mediated orofacial nociceptive information is relayed directly to the postsynaptic neurons whereas in the Vc, it is presynaptically modulated for a considerable number of TRPV1+ boutons before transmission to the postsynaptic neurons, and ultimately that it may be processed differently in the LPBN than in the Vc.

The lack of axoaxonic synapses on the TRPV1+ boutons in the LPBN is analogous to that on the boutons of peptidergic C afferents in the SDH (Knyihar-Csillik et al., 1982; Alvarez et al., 1993) but at odds with that on boutons of most primary sensory afferent types, such as non-peptidergic C afferents (Gerke and Plenderleith, 2004; Kim et al., 2008), Aδ high threshold- and Aβ low threshold-mechanoreceptive afferents (Alvarez et al., 1992, 1993; Moon et al., 2008), which frequently receive axoaxonic synapses from GABA + presynaptic axon terminals in the Vc and SDH. Considering together with (1) lack of axoaxonic synapses on the TRPV1+ boutons in the LPBN in the present study, (2) coexpression of CGRP and/or substance P in the TRPV1+ TG neurons (Bae et al., 2004) and (3) direct projection of CGRP + trigeminal afferent to the LPBN (Rodriguez et al., 2017), it is possible to assume that only peptidergic TRPV1+ trigeminal neurons may project directly to the LPBN.

## Data availability statement

The original contributions presented in this study are included in the article/supplementary material, further inquiries can be directed to the corresponding author.

## Ethics statement

The animal study was reviewed and approved by the Research and Ethics Committee of Kyungpook National University.

## Author contributions

YB: study design and writing of the manuscript. SA, YC, and SP: immunohistochemistry and electron microscopy. SA, YC, YK, and YB: analysis and interpretation of the data. All authors have had full access to all the data in this study and take responsibility for the integrity of the data and the accuracy of the analysis.
